# The influence of individual characteristics and non‐respiratory diseases on blood eosinophil count

**DOI:** 10.1002/clt2.12036

**Published:** 2021-06-03

**Authors:** Rita Amaral, Tiago Jacinto, Andrei Malinovschi, Christer Janson, David Price, João A. Fonseca, Kjell Alving

**Affiliations:** ^1^ CINTESIS—Center for Health Technology and Services Research Faculty of Medicine University of Porto Porto Portugal; ^2^ Department of Cardiovascular and Respiratory Sciences Porto Health School Polytechnic Institute of Porto Porto Portugal; ^3^ Department of Women's and Children's Health Paediatric Research Uppsala University Uppsala Sweden; ^4^ MEDCIDS‐ Department of Community Medicine, Information, and Health Sciences Faculty of Medicine University of Porto Porto Portugal; ^5^ Department of Medical Sciences Clinical Physiology Uppsala University Uppsala Sweden; ^6^ Department of Medical Sciences Respiratory, Allergy and Sleep Research Uppsala University Uppsala Sweden; ^7^ Observational and Pragmatic Research Institute Singapore Singapore; ^8^ Division of Applied Health Sciences Centre of Academic Primary Care University of Aberdeen Aberdeen UK

**Keywords:** airway inflammation, blood eosinophils, eosinophilic inflammation, respiratory diseases

## Abstract

**Background:**

Blood eosinophil (B‐Eos) count is an emerging biomarker in the management of respiratory disease but determinants of B‐Eos count besides respiratory disease are poorly described. Therefore, we aimed to evaluate the influence of non‐respiratory diseases on B‐Eos count, in comparison to the effect on two other biomarkers: fraction of exhaled nitric oxide (FeNO) and C‐reactive protein (CRP), and to identify individual characteristics associated with B‐Eos count in healthy controls.

**Methods:**

Children/adolescents (<18 years) and adults with complete B‐Eos data from the US National Health and Nutritional Examination Surveys 2005–2016 were included, and they were divided into having respiratory diseases (*n* = 3333 and *n* = 7,894, respectively) or not having respiratory disease (*n* = 8944 and *n* = 15,010, respectively). After excluding any respiratory disease, the association between B‐Eos count, FeNO or CRP, and non‐respiratory diseases was analyzed in multivariate models and multicollinearity was tested. After excluding also non‐respiratory diseases independently associated with B‐Eos count (giving healthy controls; 8944 children/adolescents and 5667 adults), the independent association between individual characteristics and B‐Eos count was analyzed.

**Results:**

In adults, metabolic syndrome, heart disease or stroke was independently associated with higher B‐Eos count (12%, 13%, and 15%, respectively), whereas no associations were found with FeNO or CRP. In healthy controls, male sex or being obese was associated with higher B‐Eos counts, both in children/adolescents (15% and 3% higher, respectively) and adults (14% and 19% higher, respectively) (*p* < 0.01 all). A significant influence of race/ethnicity was also noted, and current smokers had 17% higher B‐Eos count than never smokers (*p* < 0.001).

**Conclusions:**

Non‐respiratory diseases influence B‐Eos count but not FeNO or CRP. Male sex, obesity, certain races/ethnicities, and current smoking are individual characteristics or exposures that are associated with higher B‐Eos counts. All these factors should be considered when using B‐Eos count in the management of respiratory disease.

## 1 INTRODUCTION

Airway type‐2 inflammation is a feature of common phenotypes of asthma^1^, ^2^, ^3^ and chronic obstructive pulmonary disease (COPD).^4^, ^5^ The most studied biomarkers used to characterize patients with type‐2 inflammation are blood eosinophil (B‐Eos) count and fraction of exhaled nitric oxide (FeNO).^6^

In asthma, elevated B‐Eos count is associated with poor disease control, accelerated lung function decline, increased risk of severe exacerbations, and re‐hospitalizations.^7^, ^8^, ^9^, ^10^, ^11^ Also, it has been reported a dose‐response effect between inhaled corticosteroids (ICS) and the reduction in B‐Eos levels.^12^ In COPD patients, higher B‐Eos levels are associated with an improved response to ICS in preventing exacerbations.^13^, ^14^, ^15^, ^16^ During exacerbations, higher B‐Eos levels predict greater response to oral corticosteroids, while lower levels are associated with worse outcomes.^17^, ^18^ Furthermore, exacerbations are associated with an increased decline in lung function among COPD patients with elevated B‐Eos and without ICS.^18^ However, the overall reported odds ratios are low, and since B‐Eos count could be significantly influenced by different co‐factors, we suggest that the association between B‐Eos count and respiratory morbidity could be improved by adjusting for these factors.

In asthma, persistent type‐2 inflammation indicated by, for example, elevated FeNO or B‐Eos count, may identify patients with poor responsiveness to ICS, despite adherence to treatment.^19^ Furthermore, biomarker‐directed risk stratification to identify patients suitable for different biological treatments, including FeNO and B‐Eos count, have been proposed,^20^, ^21^ and this combination of biomarkers seems to provide additive predictive information on asthma morbidity and risk.^22^, ^23^

FeNO is affected by individual factors such as age, height and sex,^24^, ^25^ and cigarette smoke exposure,^26^ but very few studies have analyzed possible determinants of B‐Eos count. Nevertheless, individual characteristics may also affect B‐Eos values, which could hamper the clinical interpretation of a B‐Eos count, especially in the initial assessment of patients with respiratory symptoms. Elevated B‐Eos levels have been described to be associated with smoking^27^, ^28^, ^29^ and increasing age.^27^, ^29^, ^30^ However, evidence on the influence of individual factors on B‐Eos count is inconsistent, and even less data exists for subjects without respiratory disease.^27^ Moreover, there is a great controversy regarding the optimal B‐Eos cutoff that is more strongly associated with various disease outcomes, which could make the interpretation of a B‐Eos count inconsistent.^31^

Identification of factors not related to respiratory disease that should be considered when interpreting a B‐Eos count may be useful for a targeted and personalized approach in the clinical management of respiratory diseases. Moreover, in addition to the type of inflammation, its location is also important for the disease assessment. To this end, it may be necessary to have a comparison with other systemic markers and local type‐2 markers, such as C‐reactive protein (CRP) and FeNO, respectively.^32^, ^33^

The aim of this investigation was to (a) evaluate the influence of non‐respiratory disease on B‐Eos count, FeNO, and CRP, and (b) to identify individual characteristics that are associated with B‐Eos count in healthy individuals.

## 2 METHODS

### 2.1 Data source and study subjects

We analyzed the data publicly available from six 2‐year surveys (2005–2016) of the National Health and Nutritional Examination Surveys (NHANES), representing a total of 60,936 individuals from 0 to 85 years old. NHANES is a continuous nationally representative cross‐sectional survey of civilian noninstitutionalized persons in the United States of America. Detailed information can be found elsewhere.^34^ The National Center for Health Statistics Research Ethics Review Board approved the protocols (ERB protocols numbers #2004‐2005, #2006‐2007, #2008‐2010 and #2011‐2017) and the participants provided written informed consent.

### 2.2 Inflammatory biomarkers

B‐Eos count was analyzed using a quantitative hematologic analyzer and leukocyte differential cell counter, Beckman Coulter HMX (Beckman Coulter). Complete details on blood collection procedures, quality assurance, and control procedures are described elsewhere.^35^

FeNO was measured following the ATS/ERS recommendations^36^ using a handheld device with an electrochemical sensor, NIOX MINO (Aerocrine). FeNO measurements not fulfilling ATS/ERS recommendations were excluded (*n* = 6178%; 28%). Please see Additional file 1 for detailed exclusion criteria.

Serum CRP was quantified by latex‐enhanced nephelometry using a Dade Behring Nephelometer II Analyzer System (Dade Behring Diagnostics Inc.). Further details can be found elsewhere.^35^

FeNO measurements are currently available only for NHANES survey years 2007–2012 (*n* = 19,800), serum CRP is available only in survey years 2005–2010 (*n* = 23,680), whereas B‐Eos counts were measured during all included surveys (2005–2016; *n* = 49,992). Thus, all three markers were available in the 2007–2010 survey years (*n* = 20,686).

### 2.3 Variables

Demographic characteristics, such as age, sex, race/ethnicity, body mass index (BMI), and smoking status were analyzed. Two age groups were defined: *children/adolescents* (if < 18 years) and *adults* (≥18 years).

*Race/Ethnicity* was categorized as non‐Hispanic white, non‐Hispanic black, Mexican/Hispanic, and other (multi‐racial). *BMI* was categorized according to international recommendations, both for children/adolescents^37^ and adults,^38^ into underweight, normal weight, overweight, and obese. *Smoking status* was defined as: never smokers, current smokers, and former smokers. Children/adolescents were considered as nonsmokers (see Data S1 for details).

*Respiratory diseases* were considered when having a self‐reported diagnosis of asthma and/or hay fever (in children/adolescents), or self‐reported diagnosis of asthma and/or hay fever, and/or other respiratory diseases (in adults). Asthma was defined as an affirmative response to the question “Has a doctor ever told you that you had asthma?” Hay fever was defined if the subject answered positively to either: “Has a doctor ever told you that you had hay fever?” or “During the past 12 months, have you had an episode of hay fever?” Other respiratory diseases were defined if the subject answered positively to “Has a doctor or other health professional told you that you had emphysema and/or chronic bronchitis?”

*Healthy control groups* were obtained by excluding children/adolescents with any of the respiratory diseases described above. In adults, healthy controls were obtained by excluding subjects having: any respiratory disease and at least one of the non‐respiratory diseases (arthritis, heart diseases, stroke, cancer, hypertension, diabetes, hypercholesterolemia, and metabolic syndrome) that were significantly associated with B‐Eos after adjustment for individual characteristics (see below). Details on the definition of the non‐respiratory diseases are described in the Data S1.

**FIGURE 1:**
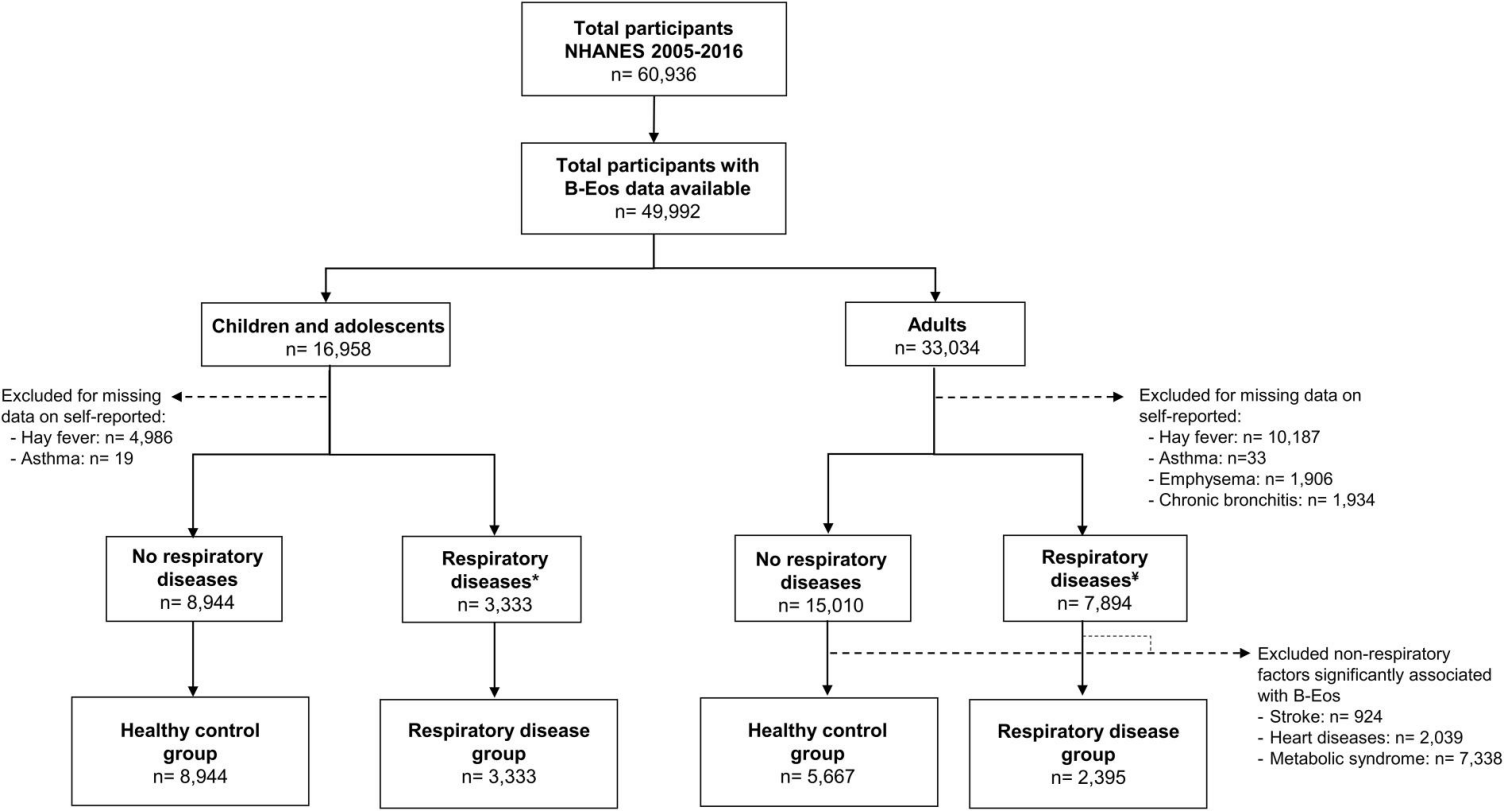
Flowchart of the National Health and Nutritional Examination Surveys (NHANES) participants. *Respiratory diseases included previous diagnosis of asthma (*n* = 1984) and/or hay fever (*n* = 1349); ¥ Respiratory diseases included previous diagnosis of asthma (*n* = 4744), hay fever (*n* = 3244) and/or other respiratory diseases (emphysema *n* = 653; chronic bronchitis: *n* = 1748)

The two *respiratory disease groups* were defined as: (a) children/adolescents with respiratory disease; and (b) adults with respiratory disease and without any significant non‐respiratory disease associated with elevated B‐Eos counts (Figure 1).

### 2.4 Statistical analysis

Statistical analyses were conducted in Stata/IC 15.1 (Stata Corp), and a statistical significance level was set at *p* < 0.05. In all analyses, the complex multistage sampling and sampling weights were considered using the *svy* package.

B‐Eos count and FeNO were log‐transformed because of their highly skewed distribution and described using geometric means and 95% confidence intervals (GM [95%CI]). Also, we categorized both B‐Eos count and FeNO measurements by using the most commonly used cut‐offs.^39^, ^40^, ^41^ Simple descriptive statistics were used to describe the study population. To explore the association between B‐Eos count and each disease factor we performed multivariate linear regression modeling. Separate univariate and multivariate models were built using each biomarker (B‐Eos, FeNO, and CRP) as the outcome variable. All variables with *p* < 0.20 were considered for inclusion in the final model. Adjustments were made for co‐variables: sex, age, race, smoking status, height, and BMI, and weight instead of BMI, in all models. Diagnostic for multicollinearity was performed using the variance inflation factor (VIF) test. A VIF above 5 was considered to indicate a high degree of correlations among the predictor variables.^42^ Coefficients (ß) with 95% confidence intervals (95% CI) were presented, and the model fit was assessed using the *svygof* function. Sensitivity analysis was performed by multiple imputation of missing values (Figure 1) using the *MI* command. Additionally, percentage change was calculated by the ratio between the amount of change and the original value, multiplied by 100.

To analyze the prevalence of the non‐respiratory disease factors, we divided age into tertiles (18–35; 36–57; ≥58 years old). Spearman correlation coefficients were calculated between B‐Eos count and other markers (FeNO and CRP), in both healthy control and respiratory disease groups (in children/adolescents and adults, respectively).

## 3 RESULTS

### 3.1 Participant characteristics

NHANES included a total of 60,936 participants from 2005 to 2016, and 82% had complete data on B‐Eos count across all ages (Figure 1). Participant characteristics for children/adolescents and adults, respectively, are described and compared in Table 1. The proportion of females was slightly higher in adults compared to the young group. Furthermore, the proportion of non‐Hispanic whites was larger among adults (Table 1). Adults reported significantly more respiratory diseases than children/adolescents, mainly due to the reporting of chronic bronchitis and emphysema as well as more hay fever, whereas the proportion reporting asthma was similar in the two age groups (Table 1). Children/adolescents showed higher B‐Eos counts (12%), whereas adults presented with higher FeNO (13%) and CRP levels (150%).

**TABLE 1:** Characteristics of the participants with B‐Eos data available in both age groups and respective comparisons

	Children/adolescents	Adults	*p*‐value
Total^a^	16,958 (21)	33,034 (79)	<0.001
Female, *n* (%)	8242 (49)	16,999 (52)	0.024
Age, mean (sd)	9.7 (4.8)	46.4 (17.4)	<0.001
BMI, mean (sd)	20.2 (5.4)	28.8 (6.8)	<0.001
BMI, *n* (%)			
Underweight	399 (3)	564 (2)	0.319
Normal	9796 (64)	9557 (30)	<0.001
Overweight	2502 (16)	10,549 (33)	<0.001
Obesity	3025 (18)	11,924 (36)	<0.001
Race/ethnicity, *n* (%)			
Non‐Hispanic White	4635 (54)	14,050 (68)	<0.001
Non‐Hispanic Black	4272 (14)	6970 (11)	0.041
Mexican/Hispanic	6318 (23)	8702 (14)	<0.001
Others (multiracial)	1733 (8)	3312 (7)	0.196
Smoking status, *n* (%)	N.A.		N.A.
Never smoker		17,606 (55)	
Current smoker		6549 (21)	
Former smoker		7397 (24)	
Respiratory diseases, *n* (%)	3333 (29)	7894 (37)	<0.001
Asthma	2721 (16)	4744 (15)	0.249
Hay fever	1035 (11)	3244 (17)	<0.001
Chronic bronchitis	N.A.	1748 (6)	N.A.
Emphysema		653 (2)	
Non respiratory diseases, *n* (%)		24,193 (83)	
Cancer		2856 (10)	
Arthritis		8297 (25)	
Heart diseases		2641 (7)	
Stroke	N.A.	1174 (3)	N.A.
Hypercholesterolemia		16,747 (52)	
Diabetes		10,276 (28)	
Hypertension		11,133 (31)	
Metabolic syndrome		10,140 (42)	
B‐Eos (/mm^3^), GM (95% CI)	198 (194–201)	176 (175–178)	<0.001
<300/mm^3^, *n* (%)	11,166 (68)	24,613 (74)	<0.001
300–500/mm^3^, *n* (%)	3604 (20)	6414 (20)	0.954
>500/mm^3^, *n* (%)	2188 (12)	2007 (6)	<0.001
FeNO (ppb), GM (95% CI)	11.7 (11.3–12.2)	13.2 (12.8–13.7)	<0.001
≥12 years (<12 years)			
<25 (<20) ppb, *n* [%]	13,260 [85] (2042 [85])		0.998
25–50 (20–35) ppb, *n* [%]	2121 [12] (224 [8])		0.076
> 50 (>35 ppb), *n* [%]	642 [3] (216 [7])		0.010
CRP (mg/dl), mean (sd)	0.16 (0.6)	0.40 (0.8)	<0.001
<0.1 mg/dl, *n* (%)	6432 (97)	14,857 (91)	<0.001
≥0.1 mg/dl, *n* (%)	240 (3)	1751 (9)	<0.001
Healthy control group, *n* (%)	8944 (71)	5667 (63)	<0.001

^a^ With available B‐Eos data. Categorical variables presented as absolute numbers and proportions weighted for the US population. Data available for FeNO (children/adolescents: *n* = 4855; adults: *n* = 13,650) and for CRP (children/adolescents; *n* = 6653; adults: *n* = 16,512).

Abbreviations: BMI, body mass index; B‐Eos, blood eosinophils levels; FeNO, fractional exhaled nitric oxide; CRP, C‐reactive protein; GM, geometric mean; CI, confidence interval. N.A. not applicable, because data were only collected in adults.

### 3.2 Effect of non‐respiratory diseases on B‐Eos count

In adults without respiratory disease (Figure 1), the prevalence of all the non‐respiratory disease factors across age tertiles is shown in Table S1. Univariate analyses showed that B‐Eos counts were higher in individuals reporting arthritis, heart disease, stroke, hypercholesterolemia, diabetes, hypertension, or metabolic syndrome than in subjects without these disorders (Table 2). Similarly, participants reporting these diseases had higher FeNO levels (except for stroke), and higher CRP levels (except for hypercholesterolemia).

**TABLE 2:** Univariate comparisons of the non‐respiratory diseases for B‐Eos, FeNO and CRP levels, in adults

	B‐Eos count (/mm^3^) *n* = 15,010		FeNO (ppb) *n* = 6700		CRP (mg/dl) *n* = 11,368	
Non‐respiratory diseases:	Geom mean (95% CI)	*p*‐value	Geom mean (95% CI)	*p*‐value	Mean (sd)	*p*‐value
Cancer					
With	176 (169–184)	0.10	14.4 (13.5–15.2)	**<0.001**	0.45 (0.9)	0.06
Without	170 (167–172)		12.7 (12.2–13.1)		0.37 (0.7)	
Arthritis						
With	176 (172–180)	**<0.001**	13.7 (13.2–14.3)	**<0.001**	0.50 (1.0)	**<0.001**
Without	168 (166–171)		12.6 (12.1–13.0)		0.35 (0.6)	
Heart diseases						
With	191 (184–198)	**<0.001**	15.0 (13.6–16.5)	**<0.001**	0.50 (1.0)	**<0.001**
Without	169 (167–171)		12.7 (12.3–13.1)		0.37 (0.7)	
Stroke						
With	194 (183–207)	**<0.001**	12.6 (11.1–14.4)	0.87	0.58 (1.1)	**<0.001**
Without	170 (167–172)		12.8 (12.4–13.2)		0.37 (0.7)	
Hypercholesterolemia						
With	172 (169–175)	**<0.001**	13.2 (12.8–13.7)	**<0.001**	0.38 (0.8)	0.61
Without	168 (165–171)		12.4 (11.9–12.9)		0.37 (0.7)	
Diabetes						
With	180 (176–183)	**<0.001**	14.2 (13.7–14.7)	**<0.001**	0.45 (0.8)	**<0.001**
Without	167 (164–169)		12.3 (11.9–12.7)		0.35 (0.7)	
Hypertension						
With	179 (175–183)	**<0.001**	14.1 (13.5–14.6)	**<0.001**	0.47 (0.8)	**<0.001**
Without	166 (164–168)		12.2 (11.7–12.6)		0.34 (0.7)	
Metabolic syndrome						
With	182 (178–186)	**<0.001**	13.8 (13.3–14.3)	**<0.001**	0.47 (0.8)	**<0.001**
Without	163 (161–166)		12.6 (12.0–13.1)		0.27 (0.6)	

*Note:* Bold values denote statistical significance at the p < 0.05 level.

Abbreviations: B‐Eos, blood eosinophil; CRP, C‐reactive protein; FeNO, fractional exhaled nitric oxide.

**FIGURE 2:**
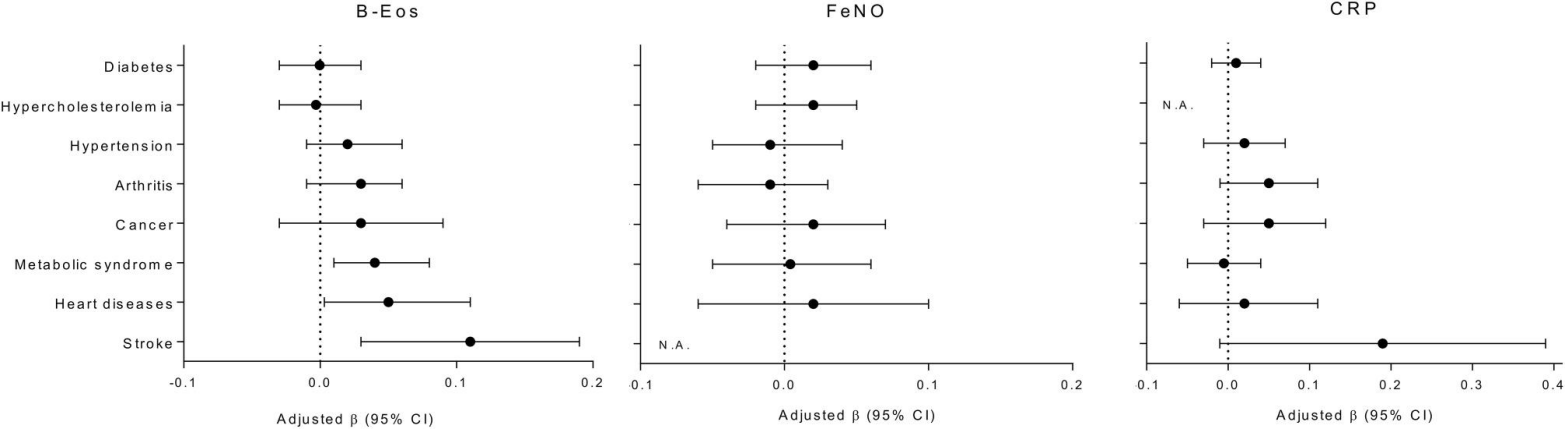
Regression coefficients (ß) and 95% confidence interval of the non‐respiratory disease factors for blood eosinophil (B‐Eos), fraction of exhaled nitric oxide (FeNO) and C‐reactive protein (CRP), in adults without respiratory diseases. Multivariate analysis adjusted for age, sex, smoking status, race, body mass index (BMI), and height. Reference category was absence of the disease. N.A. Not applicable because *p* > 0.20 in univariate analysis

After multivariable adjustment, reporting heart disease, stroke, and/or metabolic syndrome were associated with elevated B‐Eos counts, independently of individual characteristics (age, sex, smoking status, race/ethnicity, BMI, and height; Figure 2). B‐Eos count was 13%, 15%, and 12% higher, respectively, in subjects with any of these non‐respiratory diseases compared to subjects without. Using the same variables in the model, that is, both individual characteristics and reported non‐respiratory diseases, no independent associations were found between having a non‐respiratory disease, and FeNO or CRP levels. Furthermore, there was no evidence of multicollinearity among covariates in any model (maximum VIF<2.0).

### 3.3 Effect of individual characteristics on B‐Eos count in healthy control groups

After excluding children/adolescents with any respiratory disease, and adults with any respiratory disease and at least one non‐respiratory disease independently associated with B‐Eos count (heart diseases, stroke, and/or metabolic syndrome), we obtained healthy control groups with children/adolescents (*n* = 8944) and adults (*n* = 5667), respectively (Figure 1).

**FIGURE 3:**
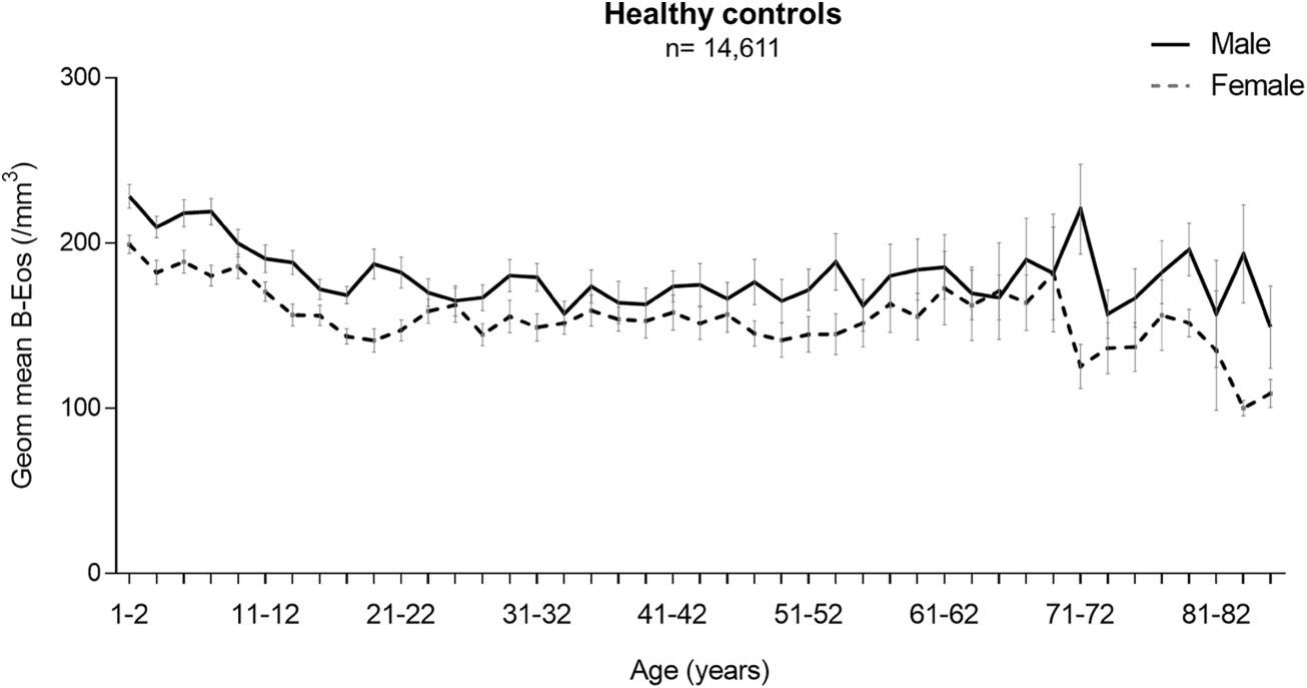
Distribution of blood eosinophil (B‐Eos) count according to age and sex in the combined healthy control groups. Geometric mean and 95% confidence intervals are presented

The overall distribution of B‐Eos count in all the participants of the healthy control groups, ranging from 1 to 85 years and stratified by sex, is illustrated in Figure 3. Males show higher B‐Eos counts compared to females across the whole age range. A decrease in B‐Eos count was seen with increasing age in individuals <18 years, followed by a stabilization up to around 70 years, regardless of sex. Furthermore, children/adolescents had significantly higher B‐Eos counts than adults regardless of individual characteristics such as sex, BMI, and race/ethnicity, except in obese individuals (Additional file 1: Table S2).

**FIGURE 4:**
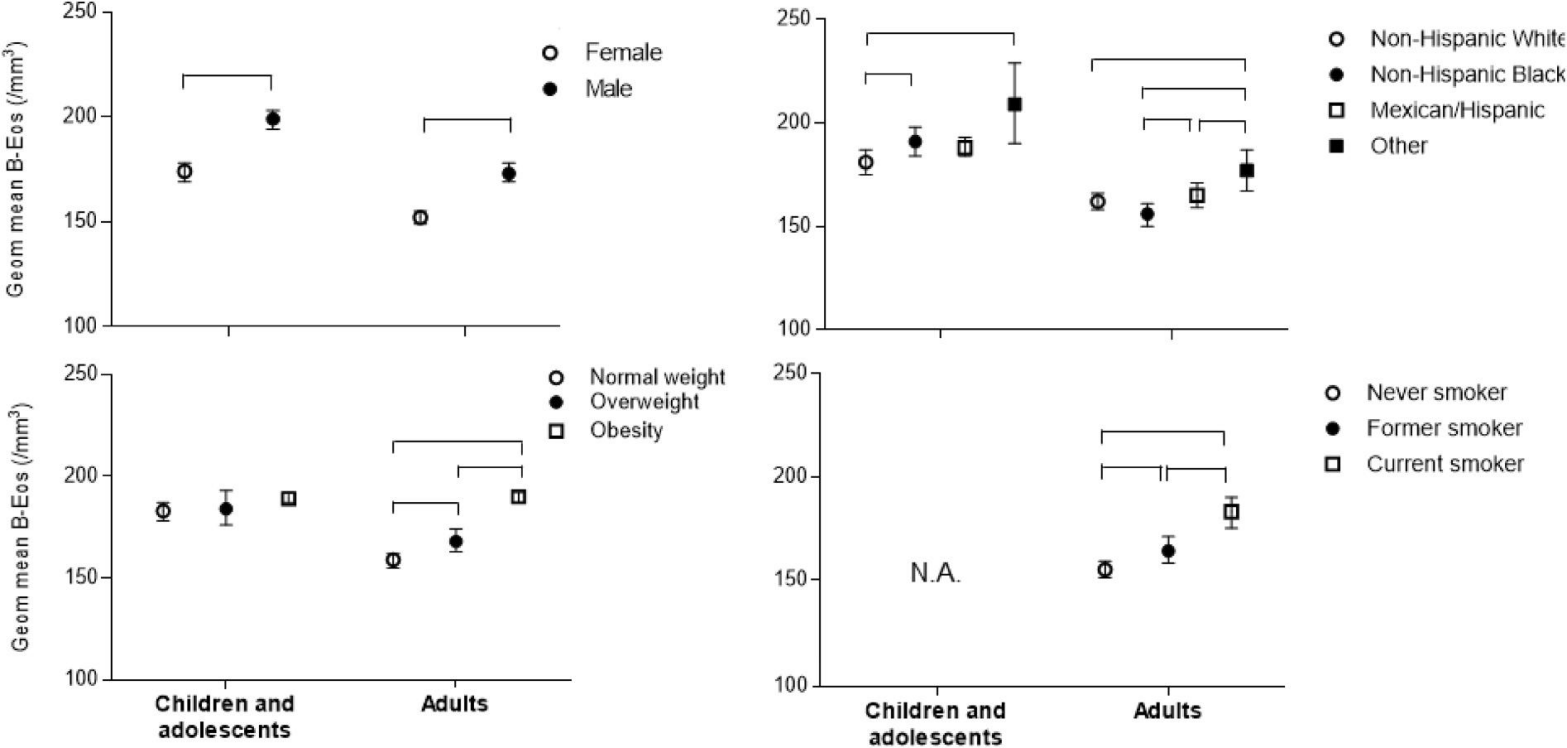
Univariate comparisons of blood eosinophil (B‐Eos) levels according to the individual characteristics of the two healthy control groups stratified by age. Horizontal brackets are the significant differences between the categories (e.g., males vs. females). Geometric mean and 95% confidence intervals are presented. N.A. Not applicable, because smoking status was only collected in adults. Subjects with respiratory diseases, heart diseases, stroke, and/or metabolic disease were excluded in all groups

In univariate analyses, males had significantly higher B‐Eos count than females among both children/adolescents and adults when analyzing absolute (Figure 4) and relative (Table 3) differences. Furthermore, differences in B‐Eos counts were seen between different groups of race/ethnicity; the most prominent difference was between non‐Hispanic whites and other race/ethnicity. In adults, being overweight or obese and being former or current smoker was associated with higher B‐Eos counts.

**TABLE 3:** Univariate and multivariate analyses of the individual factors associated with relative changes in B‐Eos count, in the healthy control groups

	Children/adolescents			Adults		
	% change	*p*‐value	Adjusted *p*‐value^a^	% change	*p*‐value	Adjusted *p*‐value^b^
Sex						
Female (ref.)						
Male	+15%	<0.001	<0.001	+14%	<0.001	<0.001
BMI						
Normal (ref.)						
Overweight	+1%	0.72	0.115	+6%	0.005	0.003
Obese	+3%	0.23	0.008	+19%	0.013	<0.001
Race/ethnicity						
Non‐Hispanic White (ref.)						
Non‐Hispanic Black	+6%	0.017	0.019	−4%	0.051	0.019
Mexican/Hispanic	+4%	0.050	0.49	+2%	0.37	0.76
Other	+15%	0.010	0.006	+9%	0.009	0.001
Smoking status						
Never smoker (ref.)	NA					
Former smoker	NA			+6%	<0.001	0.10
Current smoker	NA			+17%	<0.001	<0.001

Abbreviations: B‐Eos, blood eosinophil; BMI, body mass index.

^a^ Model adjusted for age, sex, BMI, height, and race/ethnicity.

^b^ Model adjusted for age, sex, BMI, height, race/ethnicity, and smoking status. ref.: reference group. N.A. Not applicable, because smoking status was only collected in adults.

In multivariate analyses, male sex, age (*p* = 0.025; *p* < 0.001 in 0–11 years and *p* = 0.041 in 12–17 years), being obese and being of non‐Hispanic black or other race/ethnicity, were all independently associated with higher B‐Eos counts in children/adolescents (Table 3). In adults, the same factors, except age, were independently associated with higher B‐Eos count, and being overweight or current smoker were also independently associated with higher B‐Eos counts. Furthermore, non‐Hispanic blacks showed lower B‐Eos count than non‐Hispanic whites in adults. Height was a nonsignificant factor in both age groups, and similar results were obtained using weight instead of BMI in all models (data not shown).

Of note, in adults where only respiratory diseases had been excluded (*n* = 15,010; see Figure 1), age was independently associated with higher B‐Eos count (*p* < 0.001). When introducing heart diseases, stroke, and metabolic syndrome as independent factors in this model, the age effect was no longer significant (*p* = 0.290).

**FIGURE 5:**
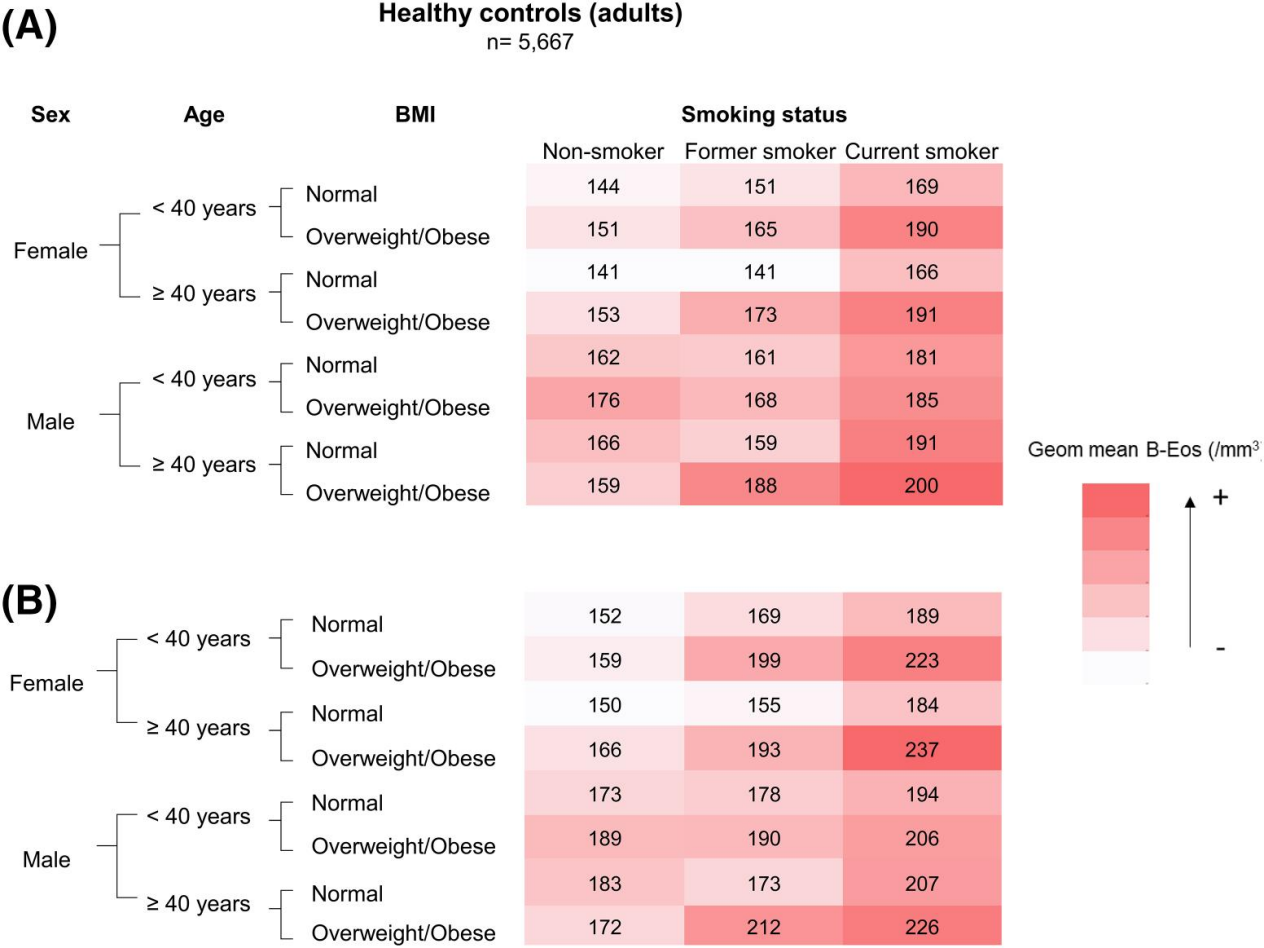
Heatmap of the geometric mean (A) and the upper bound of the 95% confidence interval (B) of B‐Eos count, according to specific individual characteristics in the adult healthy control group. Values presented in cell/mm^3^. BMI, body mass index

Figure 5 illustrates that B‐Eos count presents with a wide range of values in healthy adults, depending on different combinations of individual characteristics or exposures. As an example, females more than 40 years old, having normal weight and being never smokers, have a geometric mean of 141/mm^3^, whereas current smoking males with overweight/obesity in the same age range have a geometric mean of 200/mm^3^.

### 3.4 Association between inflammatory markers

In both children/adolescents and adults, B‐Eos count and FeNO were higher in those with respiratory disease, while CRP levels were not significantly different in the two age groups (Additional file 1: Table S3).

B‐Eos count correlated weakly with CRP in adults, both in the healthy control group and the respiratory disease group, whereas no correlation was found in children/adolescents (Additional file 1: Table S4). On other hand, FeNO correlated moderately with B‐Eos count in children/adolescents and weakly in adults, both in the healthy control group and the respiratory disease group. In adults these correlations became stronger after excluding current smokers (Additional file 1: Table S4) but was not further modified by excluding also those using ICS treatment, neither in adults nor children/adolescents (data not shown). FeNO and CRP did not correlate with each other in any group (*r* = −0.04–0.06, *p* > 0.05 all).

## 4 DISCUSSION

In a large population‐based study of US participants without respiratory disease we found that having heart disease, stroke, and/or metabolic syndrome independently increased B‐Eos levels by 12%–15%, after adjusting for covariables. No significant association was found between any non‐respiratory disease and FeNO or CRP. Furthermore, sex, overweight/obesity, race/ethnicity, and smoking status were also related to B‐Eos count. Age was independently associated with B‐Eos count only in children and adolescents.

To the best of our knowledge, this is the first study to explore the effects of non‐disease‐related factors and non‐respiratory diseases separately, performed in a large multiethnic population‐based sample. Furthermore, we compared B‐Eos count with FeNO and CRP regarding the association with non‐respiratory disease.

Eosinophils play a major role in mediating allergic inflammation. However, they have also been implicated in many other disorders, such as specific organ damage in localized infiltrative eosinophilic entities.^43^, ^44^, ^45^ In our study, having heart disease, stroke, and/or metabolic disease were independently associated with B‐Eos count in adults, similar to previous findings.^27^ This indicates that other diseases than respiratory diseases must be considered when using B‐Eos count in the management of asthma and COPD.

In spite of the correlation with B‐Eos and being well‐known and widely used in population‐based studies as a marker of systemic inflammation, CRP did not independently associate with non‐respiratory diseases in our study, whereas B‐Eos count did. This suggests that an increased level of B‐Eos may better reflect the total systemic burden of inflammation.^46^, ^47^ Although elevated CRP levels have been shown to be associated with metabolic syndrome,^48^, ^49^ and to be an independent risk factor for coronary artery disease,^50^, ^51^ CRP was in those studies not analyzed independently of individual factors such as BMI, as was done in our study.

FeNO correlated moderately with B‐Eos count but there was a significant independent association between non‐respiratory diseases and B‐Eos count only, and not FeNO. This suggests that, even though these two biomarkers are associated with type‐2 inflammation, they partly reflect separate inflammatory pathways, with FeNO being a local and B‐Eos count a systemic type‐2 marker.^22^, ^52^ Furthermore, FeNO did not correlate with CRP whereas there was a correlation between B‐Eos and CRP. This indicates that B‐Eos count but not FeNO is affected by general systemic inflammation.

The combination of B‐Eos count and FeNO could be useful to assess different aspects of airway inflammation and to identify patients suitable for different biological agents.^53^ A striking example of this is that B‐Eos count is greatly reduced by treatment with anti‐interleukin‐5 monoclonal antibodies without any noteworthy effect on FeNO.^54^, ^55^ This might indicate that personalized cutoff values for both B‐Eos and FeNO, adjusting for individual characteristics, could provide improved prognostic and predictive information.

Regarding the effect of individual characteristics on B‐Eos count, elevated B‐Eos levels have previously been described in males,^27^, ^29^ current smoking^27^, ^28^ and increasing age.^29^, ^30^ Although we obtained very similar results regarding sex and smoking status, age was independently associated with B‐Eos count only in children and adolescents. In adults, there is an association only if significant non‐respiratory diseases are not excluded. After we adjust for non‐respiratory disease factors and confirm the absence of high correlations among predictor variables, age was no longer a significant determinant of B‐Eos count in adults. There was an increase in the prevalence of non‐respiratory disease with increasing age, corroborating that there was a relationship but no co‐linearity between non‐respiratory diseases and age. These results suggests that the age effect seen in previous studies was related to non‐respiratory diseases commonly seen in middle‐aged and older adults. Our results are also consistent with observations in a recent study that analyzed a different population setting.^27^ The same authors also found a similar age trend in children and adolescents, namely that subjects in early life (≤12 years) presented the highest B‐Eos counts with an increasing trend with decreasing age, regardless of sex. The reason for higher B‐Eos counts in children should be studied further.

Eosinophilic inflammation has also been found to be associated with higher BMI among adults.^56^ In our study, obese subjects had higher B‐Eos values than those with normal weight. Also, current smoking was independently associated with higher B‐Eos levels, compared to never and former smokers. This is in line with several studies that demonstrated a significant increase in B‐Eos counts by smoke exposure.^57^ Moreover, an association between higher B‐Eos counts and serum cotinine levels was previously reported in healthy subjects, and even passive smoke exposure was shown to cause elevated B‐Eos counts.^26^ In the same study, B‐Eos count was higher in presently non‐exposed (serum cotinine below lower limit of detection) former smokers compared to never smokers. However, this could be explained by the lack of adjustment for non‐respiratory diseases and BMI.

To our knowledge, the effect of ethnicity on B‐Eos count is poorly evaluated in the literature. In our study, we found a race/ethnicity influence among healthy control groups. Specifically, non‐Hispanic white children and adolescents, and non‐Hispanic black adults presented with the lowest B‐Eos levels. However, further studies are needed to explore these results.

Our estimated B‐Eos levels of the adult healthy control group were similar to those recently obtained in the same setting,^29^ and in other populations without respiratory diseases,^58^, ^59^ but higher than those presented in the Hartl et al. study.^27^ The latter study included subjects from a different ethnical setting than in our study, and differences in B‐Eos count may also be explained by differences in the cell counting methodology.

The strengths of this study were a large number of participants, the inclusion of a broad age range and different races/ethnicities, and that we used B‐Eos as a continuous value, rather than using predefined cutoffs or median as in a previous study.^27^ Furthermore, we were able to study the effects of individual characteristics, respiratory disease, and non‐respiratory disease separately by forming the corresponding subgroups. We also used other clinically used inflammatory markers as a benchmark when studying associations with non‐respiratory diseases.

The study also has some limitations. The cross‐sectional design of the study, and the fact that most of the diseases were self‐reported, may have limited the ability to support the predictive properties of the markers. However, we used broad definitions of respiratory disease to reduce the risk of including individuals with true disease in healthy control groups. Moreover, the definitions of respiratory disease that we applied have been commonly used in NHANES reports.^60^, ^61^ However, further cross‐sectional and/or longitudinal studies that include a medical diagnosis of the analyzed respiratory diseases, including, for example, lung function tests, are needed. Also, the lack of data regarding atopy, allergic sensitization, nasal polyps, urticaria, parasitic infection, inflammatory bowel diseases, eosinophilic drug reactions and circadian variation prevented the adjustments for these variables. Although this is out of scope of our study aim, it should be further explored.

In conclusion, several individual characteristics, and non‐respiratory diseases, should be considered when interpreting B‐Eos counts. Although FeNO has long been recognized to be influenced by several individual factors, this marker did not associate with any non‐respiratory disease. The individual factors that were found to influence B‐Eos count are readily available in the clinic and could be incorporated into novel reference equations to obtain individualized cutoffs that would support a targeted and personalized approach in the clinical management of chronic respiratory diseases. With the development of new biologics that target eosinophilic airway inflammation, easy‐to‐use biomarkers such as the B‐Eos count need to be better characterized to reliably predict treatment responsiveness.

## CONFLICTS OF INTEREST

Kjell Alving has received research material from Hemocue and Thermo Fisher Scientific; payment for lectures and writing engagements from Sanofi, Circassia. David Price has board membership with AstraZeneca, Boehringer Ingelheim, Chiesi, Mylan, Novartis, Regeneron Pharmaceuticals, Sanofi Genzyme, Thermofisher; consultancy agreements with Airway Vista Secretariat, AstraZeneca, Boehringer Ingelheim, Chiesi, EPG Communication Holdings Ltd, FIECON Ltd, Fieldwork International, GlaxoSmithKline, Mylan, Mundipharma, Novartis, OM Pharma SA, PeerVoice, Phadia AB, Spirosure Inc, Strategic North Limited, Synapse Research Management Partners S.L., Talos Health Solutions, Theravance and WebMD Global LLC; grants and unrestricted funding for investigator‐initiated studies (conducted through Observational and Pragmatic Research Institute Pte Ltd) from AstraZeneca, Boehringer Ingelheim, Chiesi, Mylan, Novartis, Regeneron Pharmaceuticals, Respiratory Effectiveness Group, Sanofi Genzyme, Theravance and UK National Health Service; payment for lectures/speaking engagements from AstraZeneca, Boehringer Ingelheim, Chiesi, Cipla, GlaxoSmithKline, Kyorin, Mylan, Mundipharma, Novartis, Regeneron Pharmaceuticals and Sanofi Genzyme; payment for travel/accommodation/meeting expenses from AstraZeneca, Boehringer Ingelheim, Mundipharma, Mylan, Novartis, Thermofisher; stock/stock options from AKL Research and Development Ltd which produces phytopharmaceuticals; owns 74% of the social enterprise Optimum Patient Care Ltd (Australia and UK) and 92.61% of Observational and Pragmatic Research Institute Pte Ltd (Singapore); 5% shareholding in Timestamp which develops adherence monitoring technology; is peer reviewer for grant committees of the UK Efficacy and Mechanism Evaluation programme, and Health Technology Assessment; and was an expert witness for GlaxoSmithKline. João A. Fonseca reports grants and personal fees from AstraZeneca, personal fees from Novartis, grants and personal fees from Mundipharma, outside the submitted work. All other authors report no conflicts of interest pertaining to the submitted work.

## Supporting information

Supplementary Material
