# Supplementary material for: The influence of individual characteristics and non‐respiratory diseases on blood eosinophil count
Source: Clin Transl Allergy. 2021 Jun 3;11(4):e12036. doi: 10.1002/clt2.12036 (PMC8175041; doi:10.1002/clt2.12036)
Supplement: Supplementary file 1 — Supplementary Material [file CLT2-11-e12036-s001.docx]

**The influence of individual characteristics and non-respiratory diseases on blood eosinophil count**

**Running Title:** Determinants of blood eosinophil count

**Authors:** Rita Amaral, PhD^1,2,3,4^*, Tiago Jacinto, PhD^1,2^, Andrei Malinovschi, MD, PhD^5^, Christer Janson, MD, PhD^6^, David Price, FRCGP^7,8^, João A. Fonseca, MD, PhD^1,4^, Kjell Alving, PhD^3^

^1.^ CINTESIS - Center for Health Technology and Services Research, Faculty of Medicine, University of Porto, Porto, Portugal

^2.^ Department of Cardiovascular and Respiratory Sciences, Porto Health School, Polytechnic Institute of Porto, Porto, Portugal

^3.^ Department of Women’s and Children’s Health, Paediatric Research, Uppsala University, Uppsala, Sweden

^4.^ MEDCIDS‑ Department of Community Medicine, Information, and Health Sciences, Faculty of Medicine, University of Porto, Porto, Portugal.

^5.^ Department of Medical Sciences, Clinical Physiology, Uppsala University, Uppsala, Sweden.

^6.^ Department of Medical Sciences, Respiratory, Allergy and Sleep Research, Uppsala University, Uppsala, Sweden

^7^ Observational and Pragmatic Research Institute, Singapore, Singapore

^8^ Centre of Academic Primary Care, Division of Applied Health Sciences, University of Aberdeen, Aberdeen, United Kingdom

**Supplementary Material**

## Additional methods

### Variable definition

FeNO measurements were performed at the mobile examination center using the analyzer NIOX MINO® (Aerocrine, Solna, Sweden)^1^ and measurements were excluded if they were not in accordance with ATS/ERS guidelines^2^: not having two reproducible measurements at an expiratory flow rate of 50 ml s^−1^ (n=43), use of oral or inhaled steroids in the past two days (n=810), breathing problems requiring oxygen (n=155), problems taking deep breaths (n=313), strenuous exercise in the hour prior to the measurement (n=207). In addition to these exclusion criteria, we also excluded those who smoked last hour, ate nitrate-rich vegetables in the past 3 hours and had cough, cold or respiratory illness in the last 7 days (n= 986, n= 666 and n=4,080, respectively)

Up to four FeNO measurement attempts were made and the mean of two reproducible FeNO measurements (within 2 ppb if levels were <30 ppb or within 10% if levels were >30 ppb) was taken as the final result. The lower and upper detection limits of NIOX MINO are 5 and 300 ppb, respectively. If two measurements were below the limit of detection of the device (5 ppb), a value of 3.5 ppb (lower limit of detection divided by the square root of two) was used as the mean.

*Body mass index* (BMI) was calculated, and children/adolescents were classified as: underweight (if BMI < 5^th^ percentile); normal weight (5^th^- 85^th^ percentile); overweight (85^th^- 95^th^ percentile); obese (≥ 95^th^ percentile).^3^ In adults, underweight was considered if BMI < 18.5 kg/m^2^; normal weight if BMI 18.5–24.9 kg/m^2^; overweight if BMI 25.0-29.9 kg/m^2^; and obese if BMI ≥ 30 kg/m^2^.^4^

*Smoking status* was defined as: never smokers (if subjects smoked < 100 cigarettes during their lifetime); current smokers (if participants had a positive answer to both questions: “Have you smoked ≥100 cigarettes during lifetime” and “Do you now smoke cigarettes?”); and former smokers (if participants answered positively to the first question but negatively to the second one). Children/adolescents were considered as non-smokers.

The *non-respiratory disease factors* were defined as:

- *Arthritis, stroke,* and *cancer* (all types) were ascertained through the question “Has a doctor or other health professional ever told you that you had [disease]?”.
- *Heart disease* was defined as self-reported physician diagnosed congestive heart failure or coronary heart disease or angina pectoris or heart attack.
- *Hypertension* was defined if the participant reported diagnosed hypertension and taking antihypertensive medication or if participant did not report diagnosed hypertension and had systolic blood pressure ⩾140 mmHg or diastolic blood pressure ⩾90 mmHg. The use of antihypertensive medication was defined as the participant answered yes to the survey question (Are you now taking prescribed medicine for high blood pressure?). Systolic and diastolic blood pressure levels were measured three to four times by mercury sphygmomanometer using a standard protocol to reduce variability. Systolic blood pressure and diastolic blood pressure were calculated by averaging multiple measurements.
- *Diabetes* was defined as the participant answered yes to at least one of the survey questions “Doctor said you have diabetes”, “now taking insulin” and “now taking diabetic pills to lower your blood sugar?” or if participant did not report diagnosed diabetes and had fasting glucose ⩾100 mg·dL^−1^.
- *Hypercholesterolemia* was defined as total serum cholesterol ≥200 mg/dL or being on any concurrent pharmacologic lipid-lowering treatment or both. The use of therapy was based on an affirmative response to the survey question (Are you now following this advice to take prescribed medicine?).
- *Metabolic syndrome* was defined as having high waist circumference^5^ (proxy of central obesity) and at least two of the following^6^:

1. triglycerides ⩾150 mg·dL^−1^ or specific medication;
2. high-density lipoprotein cholesterol <40 mg·dL^−1^ in men, <50 mg·dL^−1^ in women or specific medication;
3. fasting glucose ⩾100 mg·dL^−1^;
4. systolic blood pressure ⩾140 mmHg, or diastolic blood pressure ⩾90 mmHg, or specific medication

## References

1. CDC; National Health and Nutrition Examination Survey. Respiratory Health ENO Procedures Manual. [www.cdc.gov/nchs/data/nhanes/nhanes_11_12/Respiratory_Health_ENO_Procedures_Manual.pdf](http://www.cdc.gov/nchs/data/nhanes/nhanes_11_12/Respiratory_Health_ENO_Procedures_Manual.pdf) Accessed September 3 ,2020.

2. ATS/ERS. ATS/ERS Recommendations for Standardized Procedures for the Online and Offline Measurement of Exhaled Lower Respiratory Nitric Oxide and Nasal Nitric Oxide. Am J Respir Crit Care Med 2005;171(8):912–3

3. de Onis M. Development of a WHO growth reference for school-aged children and adolescents. Bull World Health Organ 2007;85(09):660-667.

4. Hales CM, Fryar CD, Carroll MD, Freedman DS, Ogden CL. Trends in Obesity and Severe Obesity Prevalence in US Youth and Adults by Sex and Age, 2007-2008 to 2015-2016. JAMA 2018;319(16):1723-1725.

5. Song X, Jousilahti P, Stehouwer CD, et al. Comparison of various surrogate obesity indicators as predictors of cardiovascular mortality in four European populations. Eur J Clin Nutr 2013;67(12):1298-302.

6. Grundy SM, Cleeman JI, Daniels SR, et al. Diagnosis and management of the metabolic syndrome: an American Heart Association/National Heart, Lung, and Blood Institute Scientific Statement. Circulation 2005;112(17):2735-52.

**Table S1.** Prevalence (%) of non-respiratory disease factors in adults without non-respiratory disease stratified by age tertiles

|  | **18-35 years**  n=4,266 | **36-57 years**  n=5,468 | **≥58 years**  n=5,276 |
| --- | --- | --- | --- |
| Hypertension | 5.4 | 27.1 | 64.0 |
| Metabolic syndrome | 12.7 | 39.7 | 68.6 |
| Stroke | 0.2 | 1.3 | 7.0 |
| Heart diseases | 0.6 | 2.8 | 83.7 |
| Arthritis | 4.1 | 16.9 | 46.3 |
| Cancer | 1.5 | 5.7 | 19.7 |
| Diabetes | 13.8 | 26.9 | 42.1 |
| Hypercholesterolemia | 30.5 | 57.8 | 71.7 |

With B-Eos data available and without any respiratory disease (n=15,010)

**Table S2.** Distribution of the geometric mean (95% confidence interval) of the B-Eos count according to the individual characteristics in the two healthy control groups.

|  | **B-Eos count** | |  |
| --- | --- | --- | --- |
| **Healthy control group** | **Children/adolescents**  n=8,944 | **Adults**^†^  n=5,667 | **p-value** |
| **Total** | 186  (182-190) | 163  (160-166) | <0.001 |
| **Sex** |  |  |  |
| Female | 174  (169-178) | 152  (149-155) | <0.001 |
| Male | 199  (194-203) | 173  (169-178) | <0.001 |
| **BMI** |  |  |  |
| Normal | 183  (179-187) | 159  (155-162) | <0.001 |
| Overweigh | 184  (176-193) | 168  (163-174) | <0.001 |
| Obese | 189  (187-192) | 190  (187-192) | 0.74 |
| **Race/ethnicity** |  |  |  |
| Non-Hispanic White | 181  (175-187) | 162  (158-166) | <0.001 |
| Non-Hispanic Black | 191  (184-198) | 156  (150-161) | <0.001 |
| Mexican/Hispanic | 188  (184-193) | 165  (159-171) | <0.001 |
| Other | 209  (190-229) | 177  (167-187) | 0.002 |
| **Smoking status** |  |  |  |
| Never smoker | NA | 155  (151-159) |  |
| Current smoker | NA | 183  (175-190) |  |
| Former smoker | NA | 164  (158-171) |  |

^†^ No respiratory diseases, no Heart diseases, no Metabolic syndrome, and no Stroke

**Table S3.** Characteristics of participants in healthy control groups and respiratory disease groups stratified by age.

|  | **Healthy control group** | **Respiratory disease group** | **p-value** |  |
| --- | --- | --- | --- | --- |
| **Children/adolescents** | 8,944 (71) | 3,333 (29) |  |  |
| **Female**, n (%) | 4,490 (50) | 1,439 (45) | <0.001 |  |
| **Age**, mean (sd) | 9.4 (0.08) | 10.8 (0.12) | <0.001 |  |
| **BMI**, mean (sd) | 19.8 (0.09) | 21.3 (0.14) | <0.001 |  |
| **BMI**, n (%) |  |  | <0.001 |  |
| Underweight | 223 (3) | 66 (2) |  |  |
| Normal | 5,289 (66) | 1,802 (58) |  |  |
| Overweight | 1,241 (15) | 542 (17) |  |  |
| Obesity | 1,471 (16) | 784 (23) |  |  |
| **Race/ethnicity**, n (%) |  |  | <0.001 |  |
| Non-Hispanic White | 2,416 (55) | 941 (56) |  |  |
| Non-Hispanic Black | 2,246 (14) | 1,013 (17) |  |  |
| Mexican/Hispanic | 3,603 (24) | 1,026 (19) |  |  |
| Others (multiracial) | 679 (7) | 353 (8) |  |  |
| **B-Eos count**, GM (95% CI) | 186 (182-190) | 231 (222-240) | <0.001 |  |
| **FeNO**, GM (95% CI) | 10.9 (10.4-11.4) | 14.3 (13.3-15.3) | <0.001 |  |
| **CRP**, mean (sd) | 0.16 (0.01) | 0.16 (0.02) | 0.928 |  |
| **Adults** | 5,667 (68) | 2,395 (32) |  |  |
| **Female**, n (%) | 2,564 (47) | 1,192 (51) | 0.007 | |
| **Age**, mean (sd) | 40.3 (0.39) | 41.1 (0.51) | 0.199 | |
| **BMI**, mean (sd) | 25.2 (0.10) | 25.7 (0.18) | 0.002 | |
| **BMI**, n (%) |  |  | 0.002 | |
| Underweight | 213 (4) | 105 (4) |  | |
| Normal | 2,904 (54) | 1,184 (50) |  | |
| Overweight | 1,683 (29) | 681 (29) |  | |
| Obesity | 861 (13) | 421 (17) |  | |
| **Race/ethnicity**, n (%) |  |  | <0.001 | |
| Non-Hispanic White | 2,247 (64) | 1,223 (73) |  | |
| Non-Hispanic Black | 1,226 (12) | 490 (10) |  | |
| Mexican/Hispanic | 1,606 (16) | 410 (10) |  | |
| Other (multiracial) | 588 (9) | 272 (7) |  | |
| **Smoking status**, n (%) |  |  | 0.01 | |
| Never smoker | 3,303 (57) | 1,256 (53) |  | |
| Current smoker | 1,296 (23) | 655 (26) |  | |
| Former smoker | 1,059 (20) | 477 (21) |  | |
| **B-Eos count**, GM (95% CI) | 163 (160-166) | 186 (180-192) | <0.001 | |
| **FeNO**, GM (95% CI) | 12.3 (11.8-12.8) | 14.4 (13.4-15.5) | <0.001 | |
| **CRP**, mean (sd) | 0.27 (0.25-0.29) | 0.32 (0.27-0.40) | 0.073 | |

Categorical variables presented as absolute numbers and proportions weighted for the U.S. population. Respiratory diseases were considered as having a previous diagnosis of asthma and/or hay fever, in children and adolescents, and having a previous diagnosis of asthma, hay fever, chronic bronchitis and/or emphysema, in adults. GM, geometric mean; CI, confidence interval.

## **Table S4.** Correlation coefficients between B-Eos count, and CRP and FeNO

|  | **CRP** | | **FeNO** | |
| --- | --- | --- | --- | --- |
|  | **r** | **p-value** | **r** | **p-value** |
| **Children/adolescents** | | |  |  |
| Healthy control (n=8,944) | 0.01 | 0.66 | 0.26 | <0.001 |
| Respiratory diseases (n=3,333) | -0.02 | 0.57 | 0.37 | <0.001 |
| **Adults** |  |  |  |  |
| Healthy control (n=5,667) | 0.06 | 0.018 | 0.09 | <0.001 |
| Never/former smokers (n= 4,362) | 0.05 | 0.004 | 0.16 | <0.001 |
| Respiratory diseases (n=2,395) | 0.05 | 0.025 | 0.15 | <0.001 |
| Never/former smokers (n=1,733) | 0.06 | 0.052 | 0.26 | <0.001 |

B-Eos count and FeNO were log-transformed; r= correlation coefficients.
